# Device-related infection and mortality in patients with chronic kidney disease receiving cardiac implantable electronic devices: a propensity score-matched cohort study

**DOI:** 10.1186/s12879-023-08773-0

**Published:** 2023-11-13

**Authors:** Po-Jui Wu, Huang-Chung Chen, Yen-Nan Fang, Shaur-Zheng Chong, Yung-Lung Chen, Mien-Cheng Chen

**Affiliations:** Division of Cardiology, Department of Internal Medicine, College of Medicine, Kaohsiung Chang Gung Memorial Hospital, Chang Gung University, 123 Ta Pei Road, Niao Sung District, Kaohsiung City, 83301 Taiwan

**Keywords:** Antiseptic preparation, Cardiac implantable electronic devices, Chronic Kidney Disease, Device-related Infection, Mortality

## Abstract

**Background:**

Chronic kidney disease (CKD) was reported to be a risk factor of cardiac implantable electronic device (CIED) infection. The application of bundled skin antiseptic preparation before CIED implantation decreased the risk of CIED infection, even in patients undergoing complex procedures. However, the effect of bundled skin antiseptic preparation to prevent CIED infection in patients with CKD was not tested.

**Methods:**

Between July 2012 and December 2019, 1668 patients receiving CIEDs comprised this retrospective cohort study and were categorized into two groups by the diagnosis of CKD: group with CKD (n = 750, 45%) and group without CKD (n = 918, 55%). The primary outcome was clinical CIED infection, including major and minor infection, and the secondary outcomes were cardiovascular mortality and all-cause mortality. Propensity score matching (PSM) was applied to reduce selection bias between the study groups.

**Results:**

During a 4-year follow-up period, 30 patients (1.8%) had a CIED infection. After PSM, the incidence of CIED infection was similar between the patients with CKD and without CKD (1.0% vs. 1.8%). The incidences of cardiovascular mortality and all-cause mortality were higher in patients with CKD compared to patients without CKD (6.5% vs. 3.0%, P = 0.009; 22.8% vs. 11.8%, P < 0.001, respectively).

**Conclusion:**

The incidence of clinical CIED infection in patients with CKD was as lower as in patients without CKD after applying the bundled skin antiseptic preparation strategy. The cumulative incidences of cardiovascular mortality and all-cause mortality were significantly higher in the matched CIED recipients with CKD compared to the matched cohort without CKD.

## Introduction

Chronic kidney disease (CKD) poses a growing and serious problem to the global health of human being, and is an important risk factor for cardiovascular morbidity and mortality [1–3]. According to the Global Burden of Disease, Injuries, and Risk Factors Study, the prevalence of CKD is 9.1% of the global population in 2017 [3]. Furthermore, CKD has been a leading cause of death in the worldwide, owing to ageing and an increasing burden of risk factors for CKD, such as diabetes and hypertension [3]. Cardiac implantable electronic devices (CIEDs), including permanent pacemaker (PPM), implantable cardioverter-defibrillator (ICD) and cardiac resynchronization therapy (CRT), are effective therapy for the treatment of bradyarrhythmias, ventricular tachyarrhythmias and systolic heart failure (HF), and are increasingly used in patients with CKD and end-stage renal disease (ESRD), which are at high risk for malignant arrhythmias, coronary artery disease, or HF [4]. Prior studies reported that CIED was present in 6–10% of patients with ESRD [5, 6]. Recently, our study showed that the prevalence of CKD is up to 39.4% in patients receiving de novo PPM [7]. However, CIED infection is a critical complication of CIED implantation, resulting in substantial incremental length of hospital stay, admission cost, in-hospital mortality, and all-cause mortality [8, 9]. CKD and ESRD are two well-established and inevitable patient-related risk factors for CIED infection [6, 10–14]. In addition, CIED infection caused significantly poor outcomes in patients with CKD or ESRD compared to patients with normal renal function [15–18]. Based on our previous studies, the application of bundled skin antiseptic preparation before CIED implantation decreased the risk of CIED infection, even in patients undergoing complex procedures [19, 20]. Whether application of bundled skin antiseptic preparation before CIED implantation could decrease the risk of CIED infection in CIED recipients with CKD remains unexplored. Accordingly, we conducted this retrospective cohort study to assess and compare the incidence of CIED infection between CIED recipients with and without CKD after propensity score matching (PSM).

## Materials and methods

### Study cohort

**Fig. 1:**
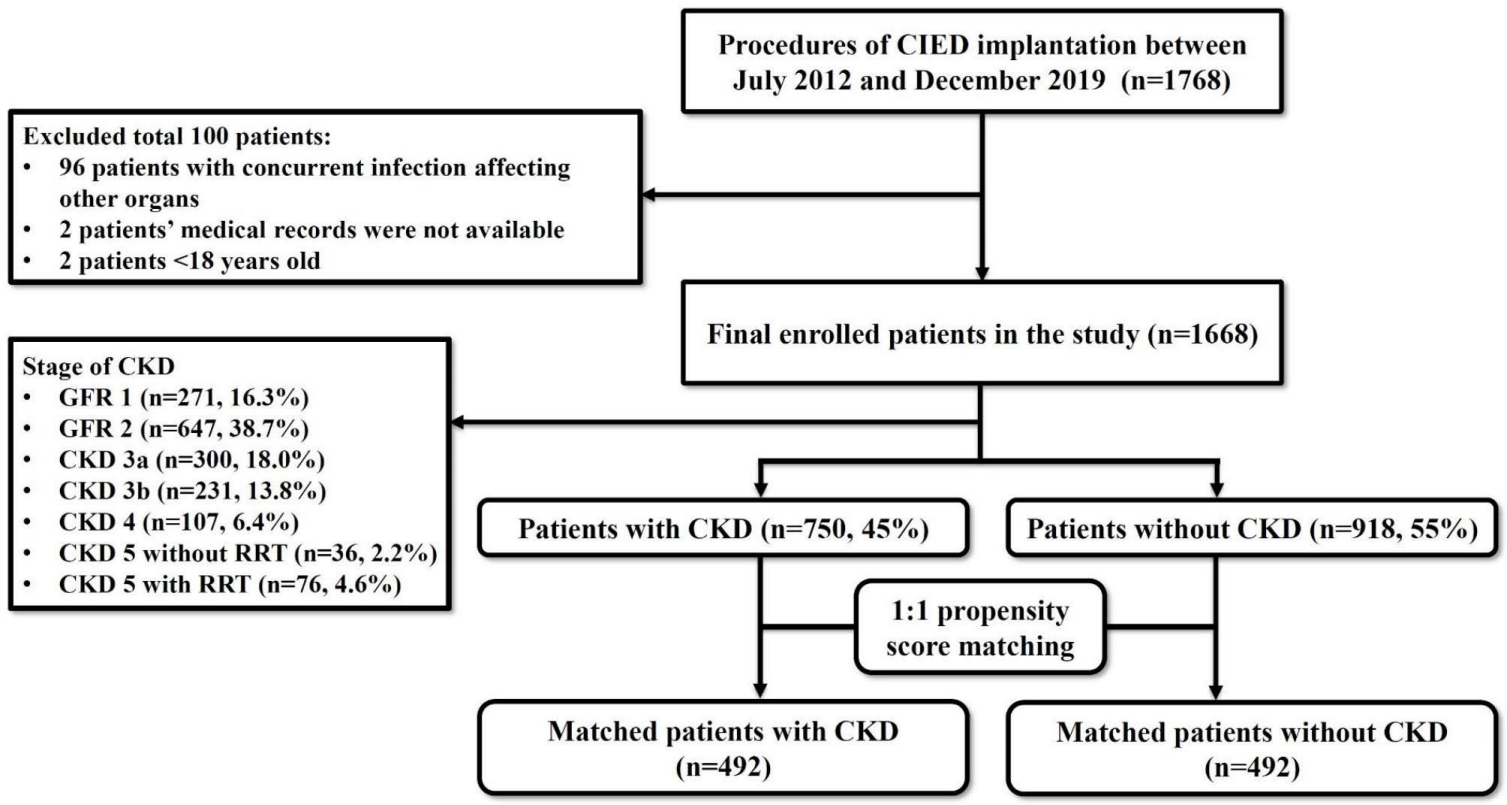
Flow chart of enrollment of patients receiving cardiac implantable electronic devices. CIED, cardiovascular implantable electronic devices; CKD, chronic kidney disease; RRT, renal replacement therapy

This retrospective cohort study enrolled 1768 consecutive patients receiving bundled skin antiseptic preparation and CIEDs implantation in our hospital between July, 2012 and December, 2019. After excluding 96 patients with concurrent infection affecting other organs, 2 patients with unavailable medical records and 2 patients younger than 18 years old, 1668 patients were enrolled (Fig. 1) and were categorized into two groups by the presence or absence of diagnosis of CKD at the time of CIED implantation: group with CKD (n = 750, 45%), and group without CKD (n = 918, 55%) (Fig. 1).

### Definitions

Based on the 2012 Kidney Disease: Improving Global Outcomes (KDIGO) Clinical Practice Guideline for the Evaluation and Management of CKD, criteria for CKD should include markers of kidney damage, such as albuminuria or urine sediment abnormalities for > 3 months and decreased glomerular filtration rate (GFR) is defined as a GFR of < 60 mL/min/1.73 m [2] for > 3 months (GFR categories G3a-G5) [21]. Estimated glomerular filtration rate (eGFR) was calculated by using the Modification of Diet in Renal Disease equation with four variables (age, gender, serum creatinine, and ethnicity) [22]. In this study, CKD is defined as a GFR of < 60 mL/min/1.73 m [2] for > 3 months (corresponding to GFR categories G3a-G5 in 2012 KDIGO guideline), and the GFR categories and CKD are as follows: GFR 1 (normal or high, GFR ≥ 90 mL/min/1.73 m [2]), GFR 2 (mildly decreased, GFR of 60–89 mL/min/1.73 m [2]), CKD 3a (mildly to moderately decreased, GFR of 45–59 mL/min/1.73 m [2]), CKD 3b (moderately to severely decreased, GFR of 30–44 mL/min/1.73 m [2]), CKD 4 (severely decreased, GFR of 15–29 mL/min/1.73 m [2]), and CKD 5 (GFR of < 15 mL/min/1.73 m [2], kidney failure with or without renal replacement therapy) [21]. GFR1 and GFR2 are classified as non-CKD. ESRD was defined as the need for renal replacement therapy including peritoneal dialysis, hemodialysis, or renal transplantation. According to the World Health Organization, anemia is defined as hemoglobin levels < 12.0 g/dL in women and < 13.0 g/dL in men [23]. Complex procedure is defined as a CIED implantation for generator replacement, ICD, CRT, and device upgrade.

### The standard protocol for CIED implantation and pre-operative bundled skin antiseptic preparation

The standard protocol for CIED implantation in our center had been described in our previous study [19]. Since July 2012, our institute applied this novel strategy of skin antiseptic preparation, named “bundled skin antiseptic preparation”, consisting of step 1: after taking a shower or bathe, the whole anterior chest wall of the patient was sterilized with a 75% alcohol solution, and then covered with large-sized sterilized gauze on the night before the procedure; step 2: 10 min prior to CIED implantation, sterile gauzes soaked in povidone-iodine (75 mg/mL) were wiped around the incision site in concentric circles, moving towards periphery, for three times and then patted dry; and step 3: finally, we applied the standard antiseptic skin preparation [19].

### Clinical outcomes

The primary outcome of this study was clinical device-related infection of patients after CIED implantation. Device-related infection was divided into major and minor infections according to clinical presentation and management. Major infection was defined as any presentation of erosive wound, bloodstream infection, pacemaker-related endocarditis, or need for surgical removal. Minor infection was defined as the local inflammatory signs including erythema, warmth, fluctuance, or tenderness at the pocket sites, presentation of any discharge, or wound dehiscence [19]. The secondary outcomes of this study included cardiovascular mortality and all-cause mortality. Cardiovascular mortality was defined as death from myocardial infarction, HF, refractory ventricular arrhythmias, or cardiac arrest. After CIED implantation, patients were followed up monthly for the first three months and then every 3–6 months until clinical outcomes of interest, death, loss to follow up, or the latest date in the dataset (30 April, 2022), whichever came first.

### Statistical analysis

Continuous variables are expressed as mean ± standard deviation or percentages. The clinical characteristics of the study groups were compared using the independent t-test for continuous variables and Chi-square test or Fisher’s exact test for categorical variables. PSM was applied to make the covariates balanced between the study groups. The variables selected to calculate propensity score were listed in Table 1. Using NCSS 10 Statistical Software (LLC, Kaysville, Utah, USA), the greedy method was used for matching at a 1:1 ratio between the study groups with a caliper width 0.2-fold of the standard deviation of the logit of the propensity score. The quality of matching was checked using the absolute value of standardized difference between the groups, where a value < 0.1 was considered negligible difference [24]. The incidences of cardiovascular mortality and all-cause mortality during long-term follow-up were expressed with Kaplan-Meier survival curves and were compared by log-rank test. The risks of time to event outcomes between groups were compared using a Cox proportional hazards model. The significance of each variable in predicting all clinical outcomes was tested using the Cox proportional hazards model, analyzed with forward option.

**Table 1:** Baseline characteristics of patients with and without chronic kidney disease before and after propensity score matching

	Before matching					After matching			
	CKD ^a^ (n = 750)	Non-CKD (n = 918)	P value	SMD		CKD ^a^ (n = 492)	Non-CKD (n = 492)	P value	SMD
**Baseline characteristics**									
Age, (years)	76 ± 10	70 ± 13	< 0.001	0.574		75 ± 10	75 ± 10	0.844	0.013
Male	371 (49.5)	497 (54.1)	0.057	0.093		247 (50.2)	264 (53.7)	0.278	0.069
Body mass index, (kg/m^2^)	25 ± 4	25 ± 4	0.139	0.074		25 ± 4	25 ± 4	0.880	0.010
Hypertension	596 (79.5)	585 (63.7)	< 0.001	0.357		366 (74.4)	373 (75.8)	0.606	0.033
Diabetes mellitus	359 (47.9)	272 (29.6)	< 0.001	0.383		190 (38.6)	197 (40.0)	0.648	0.029
Hyperlipidemia	280 (37.3)	308 (33.6)	0.108	0.081		167 (33.9)	172 (35.0)	0.737	0.021
Coronary artery disease	232 (30.9)	160 (17.4)	< 0.001	0.320		112 (22.8)	120 (24.4)	0.548	0.038
Heart failure history	220 (29.3)	205 (22.2)	0.001	0.162		136 (27.6)	124 (25.2)	0.386	0.055
Valvular heart disease ^b^	60 (8.0)	68 (7.4)	0.651	0.023		35 (7.1)	34 (6.9)	0.221	0.008
Atrial fibrillation	284 (37.9)	363 (39.5)	0.485	0.030		196 (39.8)	194 (39.4)	0.896	0.008
Cerebrovascular accident	125 (16.7)	128 (13.9)	0.123	0.080		75 (15.2)	74 (15.0)	0.940	0.006
End-stage renal disease ^c^	89 (11.9)	N/A	N/A	N/A		46 (9.4)	N/A	N/A	N/A
**Laboratory data**									
Hemoglobin, (g/dL)	11.7 ± 1.9	13.0 ± 1.8	< 0.001	0.673		12.3 ± 1.8	12.3 ± 1.8	0.785	0.017
Anemia	486 (64.8)	329 (35.8)	< 0.001	N/A		259 (52.6)	258 (52.4)	0.949	N/A
Serum creatinine, (mg/dL)	2.3 ± 2.2	0.9 ± 0.2	< 0.001	N/A		2.1 ± 2.0	0.9 ± 0.2	< 0.001	N/A
eGFR, (mL/min/1.73m^2^)	37 ± 17	84 ± 22	< 0.001	N/A		40 ± 15	82 ± 23	< 0.001	N/A
**Pacemaker procedure-related parameters**									
Transvenous temporary pacemaker ^d^	165 (22.0)	142 (15.5)	0.001	0.169		94 (19.1)	97 (19.7)	0.809	0.015
Indications for devices									
Patients with sinus nodal dysfunction	325 (43.3)	445 (48.5)	N/A	N/A		224 (45.5)	225 (45.7)	N/A	N/A
Patients with atrioventricular block	201 (26.8)	204 (22.2)	N/A	N/A		118 (24.0)	113 (23.0)	N/A	N/A
New-implant pacemaker	526 (70.1)	649 (70.7)	0.829	N/A		342 (69.5)	338 (68.7)	0.836	N/A
Complex procedure	224 (29.9)	269 (29.3)	0.802	0.008		150 (30.5)	154 (31.3)	0.783	0.022
Generator replacement	139 (18.5)	170 (18.5)		N/A		91 (18.5)	104 (21.1)		N/A
ICD	55 (7.3)	82 (8.9)		N/A		38 (7.7)	40 (8.1)		N/A
CRT	28 (3.7)	11 (1.2)		N/A		20 (4.1)	7 (1.4)		N/A
Procedures of device upgrade	2 (0.3)	6 (0.7)		N/A		1 (0.2)	3 (0.6)		N/A
Number of pacemaker lead	1.5 ± 0.8	1.5 ± 0.8	0.203	N/A		1.5 ± 0.8	1.5 ± 0.8	0.185	N/A
Pocket hematoma ^e^	22 (2.9)	27 (2.9)	0.992	< 0.001		16 (3.3)	13 (2.6)	0.572	0.036

Data are presented as mean ± SD or number (%) of patients

^a^ Defined as eGFR lower than 60 mL/min/1.73m^2^ without renal replacement therapy

^b^ Defined as moderate to severe regurgitation or stenosis of aortic, mitral or tricuspid valves

^c^ Defined as the need for peritoneal dialysis, hemodialysis, or renal transplantation

^d^ Defined as a bridge prior to permanent device implantation

^e^ Defined as a swelling and painful mass with ecchymosis formation and extending the margin of generators

CKD = chronic kidney disease; CRT = cardiac resynchronization therapy; eGFR = estimated glomerular filtration rate; ICD = implantable cardioverter-defibrillator; N/A = not applicable; SMD = standardized mean difference

Subgroup analysis was performed to evaluate the effect of bundled skin antiseptic preparation for CIED infection in subgroups of patients defined by baseline characteristics, including age (< 70, ≥ 75 years), gender, body mass index (< 27, ≥ 27), hypertension, diabetes mellitus, coronary artery disease, HF, atrial fibrillation, cerebrovascular accident, anemia, use of transvenous temporary pacemaker, complex procedure, and presentation of pocket hematoma. The P values for interactions between groups were assessed. A two-sided P value < 0.05 was considered statistically significant. All statistical analyses were performed using SPSS for Windows (version 22.0; SPSS Inc., Chicago, IL, USA) and R v3.6.1 software.

## Results

### Baseline characteristics of the study patients with and without CKD

Table 1 lists the clinical characteristics of the study patients. Before PSM, the mean age of the patients was 73 ± 12 years and 52.0% of the study patients were male. There were 918 patients without CKD and 750 patients with CKD, including CKD 3a in 300 patients, CKD 3b in 231 patients, CKD 4 in 107 patients, CKD 5 without renal replacement therapy in 36 patients and CKD 5 with renal replacement therapy in 76 patients (Fig. 1). The patients with CKD were older, and had higher prevalences of history of hypertension, diabetes mellitus, coronary artery disease, HF history, anemia, and transvenous temporary pacemaker placement compared to the patients without CKD (Table 1). The patients with CKD had lower level of hemoglobin compared to the patients without CKD (Table 1). There was no difference in the CIED procedures between the two groups.

After PSM, the baseline characteristics, except serum creatinine and eGFR, listed in Table 1 were well-balanced between the two groups. In the cohort after 1:1 PSM, 492 pairs of patients with and without CKD were analyzed.

### Clinical outcomes of the study patients before and after PSM

During a mean follow-up period of 4.2 ± 2.6 years, before PSM, the incidence of CIED infection did not differ between the patients with CKD and without CKD (0.9% vs. 2.5%, hazard ratio [HR] = 0.53, 95% confidence interval [CI], 0.22–1.30, P = 0.165) (Table 2). The incidence of major and minor CIDE infection were also similar between the two groups (0.1% vs. 0.8%, P = 0.475; 0.8% vs. 1.7%, P = 0.224, respectively) (Table 2). After PSM, the incidence of total, major and minor CIED infection still did not differ between the two groups (Table 2).

**Table 2:** Clinical outcomes of patients with and without chronic kidney disease during a 4-year follow-up period

	Before matching					After matching			
	CKD (n = 750)	Non-CKD (n = 918)	HR (95% CI)	P value		CKD (n = 492)	Non-CKD (n = 492)	HR (95% CI)	P value
**Primary outcome**									
Device-related infection	7 (0.9)	23 (2.5)	0.53 (0.22–1.30)	0.165		5 (1.0)	9 (1.8)	0.61 (0.19–1.93)	0.397
Major infection	1 (0.1)	7 (0.8)	0.41 (0.03–4.81)	0.475		0 (0)	2 (0.4)	N/A	N/A
Minor infection	6 (0.8)	16 (1.7)	0.55 (0.21–1.44)	0.224		5 (1.0)	7 (1.4)	0.61 (0.19–1.93)	0.397
**Secondary outcomes**									
Cardiovascular mortality	66 (8.8)	26 (2.8)	3.70 (2.35–5.82)	< 0.001		32 (6.5)	15 (3.0)	2.27 (1.23–4.20)	0.009
All-cause mortality	206 (27.5)	94 (10.2)	3.19 (2.50–4.08)	< 0.001		112 (22.8)	58 (11.8)	2.15 (1.49–2.81)	< 0.001

Data are presented as number (%) of patients

CI = confidence interval, HR = hazard ratio, N/A = not applicable

In the 30 patients with CIED infection, 2 patients without CKD (6.7%) had positive growth from blood cultures [caused by methicillin-susceptible staphylococcus aureus (n = 1), and stenotrophomonas maltophilia (n = 1)], and 5 patients without CKD (16.7%) had positive growth from pocket wound cultures [caused by methicillin-resistant staphylococcus aureus (n = 2), propionibacterium acnes (n = 1), enterobacter cloacae (n = 1), and achromobacter xylosoxidans (n = 1)]. In the 8 patients with major CIED infection, 7 patients (87.5%) including 1 patient with CKD and 6 patients without CKD underwent surgical removal of the pacing system for uncontrolled infection after antibiotic therapy.

**Fig. 2:**
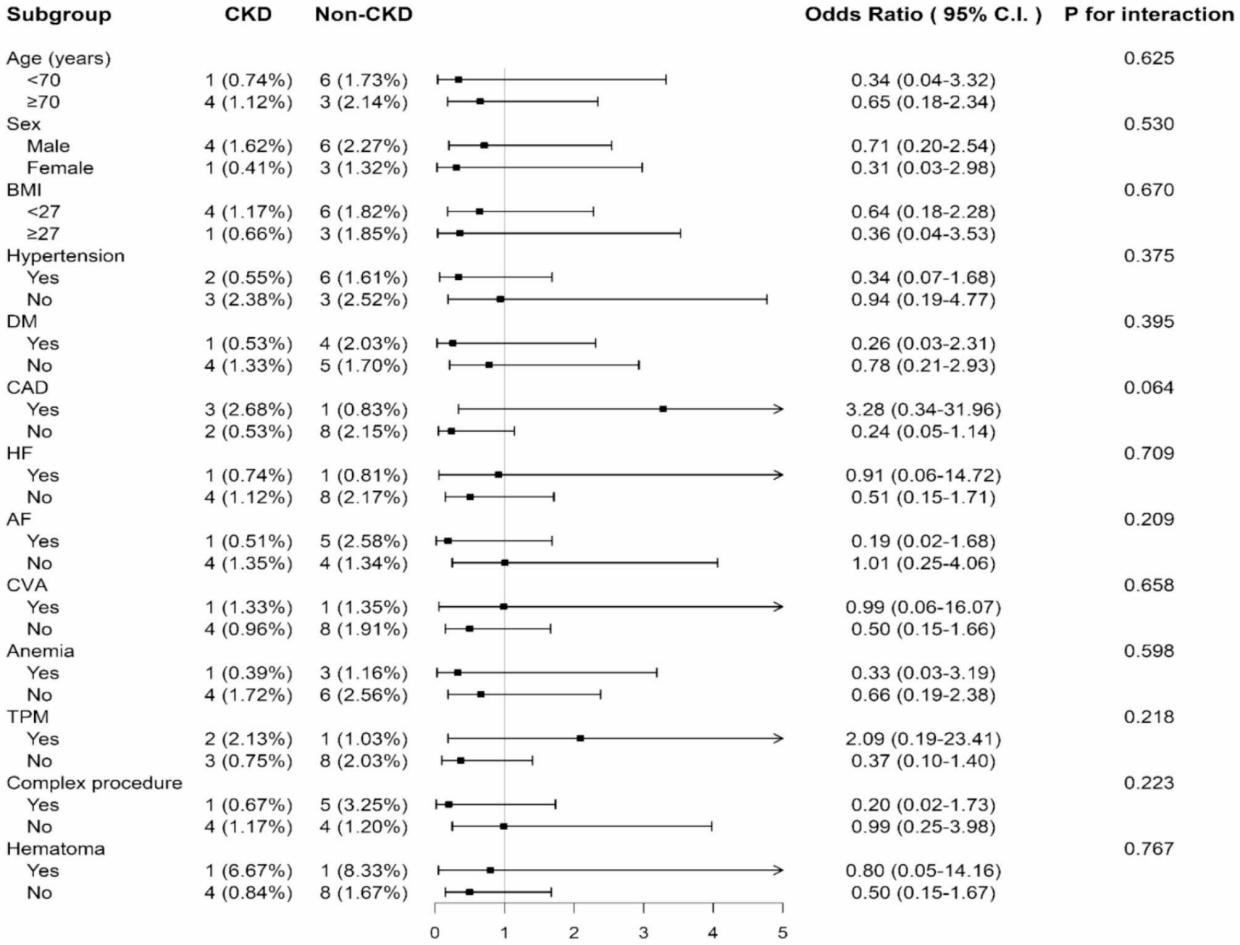
Odds ratios of clinical CIED infection between CKD and non-CKD patients among the different subgroups after applying the bundled skin antiseptic preparation strategy before surgery. AF, atrial fibrillation; BMI, body mass index; CAD, coronary artery disease; CIED, cardiac implantable electronic device; CI, confidence interval; CKD, chronic kidney disease; CVA, cerebrovascular disease; DM, diabetes mellitus; HF, heart failure; TPM, transvenous temporary pacemaker

Subgroup analysis for the primary outcome of CIED infection, the CKD group after applying the bundled skin antiseptic preparation strategy had similar incidence of CIED infection compared to the non-CKD group in different subgroups of patients in terms of age, sex, body mass index, hypertension, diabetes mellitus, coronary artery disease, HF, atrial fibrillation, cerebrovascular accident, anemia, transvenous temporary pacemaker and pocket hematoma (Fig. 2).

**Fig. 3:**
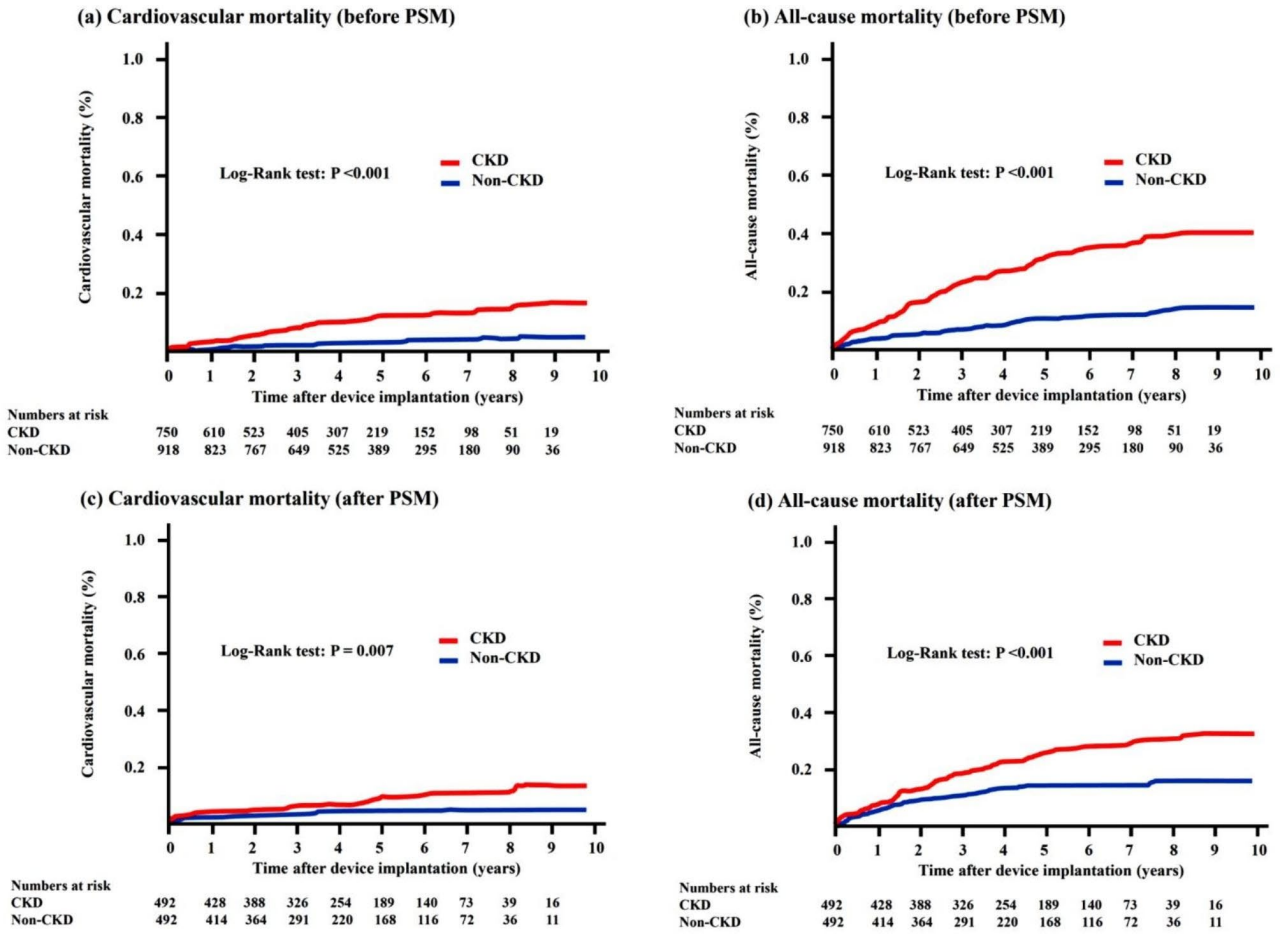
The Kaplan-Meier event-free survival curves of cardiovascular mortality and all-cause mortality between CKD and non-CKD group before (Panel **a, b**) and after (Panel **c, d**) propensity score matching. CKD, chronic kidney disease; PSM, propensity score matching

Before PSM, patients with CKD had higher incidences of cardiovascular mortality and all-cause mortality (8.8% vs. 2.8%, HR = 3.70, 95% CI, 2.35–5.82, P < 0.001; 27.5% vs. 10.2%, HR = 3.19, 95% CI, 2.50–4.08, P < 0.001, respectively) (Table 2). After PSM, the incidences of cardiovascular and all-cause mortality were still higher in patients with CKD compared to patients without CKD (6.5% vs. 3.0%, HR = 2.27, 95% CI, 1.23–4.20, P = 0.009; 22.8% vs. 11.8%, HR = 2.15, 95% CI, 1.49–2.81, P < 0.001, respectively) (Table 2). The Kaplan–Meier curve analyses for cardiovascular and all-cause mortalities before and after PSM for the two groups are shown in Fig. 3. Patients with CKD had a higher cumulative incidence of cardiovascular mortality compared to patients without CKD before and after PSM (log-rank test, P < 0.001 and P = 0.007, respectively) (Fig. 3a and c). Patients with CKD had a higher cumulative incidence of all-cause mortality compared to patients without CKD before and after PSM (log-rank test, all P < 0.001) (Fig. 3b and d).

### Determinants of CIED Infection and all-cause mortality in the matched cohort

During a 4-year follow-up period, there were 30 patients (1.8%) diagnosed with CIED infection, including 22 patients (1.3%) with minor infection and 8 patients (0.5%) with major infection (Table 2). After applying the bundled skin antiseptic preparation strategy, CKD was not a significant predictor of CIED infection before and after PSM (Table 3).

**Table 3:** Univariate and multivariate Cox regression analysis of predictors of device-related infection in the overall and matched cohorts

	Overall cohorts					Matched cohorts			
	Univariate analysis		Multivariate analysis			Univariate analysis		Multivariate analysis	
	HR (95% CI)	P value	HR (95% CI)	P value		HR (95% CI)	P value	HR (95% CI)	P value
Age, (years)	1.02 (0.98–1.06)	0.447				1.05 (0.97–1.12)	0.220		
Male	1.01 (0.45–2.31)	0.974				0.35 (0.09–1.32)	0.120	0.48 (0.11–2.07)	0.327
Body mass index, (kg/m^2^)	0.96 (0.87–1.05)	0.335				0.85 (0.70–1.02)	0.075	0.88 (0.72–1.07)	0.206
Hypertension	0.64 (0.30–1.38)	0.255				1.10 (0.36–3.33)	0.866		
Diabetes mellitus	0.75 (0.35–1.58)	0.447				0.21 (0.04–1.01)	0.051	0.43 (0.07–2.64)	0.362
Hyperlipidemia	0.67 (0.30–1.49)	0.327				1.03 (0.22–4.80)	0.970		
Coronary artery disease	1.41 (0.55–3.58)	0.476				1.05 (0.31–3.50)	0.939		
Heart failure history	0.96 (0.39–2.40)	0.934				1.22 (0.26–5.78)	0.802		
Atrial fibrillation	0.54 (0.26–1.15)	0.112	0.60 (0.27–1.31)	0.198		0.76 (0.25–2.35)	0.637		
Cerebrovascular accident	1.36 (0.54–3.42)	0.514				0.96 (0.21–4.45)	0.954		
Chronic kidney disease	1.88 (0.77–4.58)	0.165	1.56 (0.62–3.94)	0.343		1.65 (0.52–5.23)	0.397		
End-stage renal disease	1.32 (0.17–9.97)	0.789				N/A	N/A		
Anemia	1.49 (0.62–3.58)	0.378				0.82 (0.25–2.70)	0.750		
Intravenous temporary pacemaker	1.42 (0.58–3.50)	0.441				0.69 (0.18–2.59)	0.580		
Complex procedure	1.07 (0.47–2.45)	0.868				2.24 (0.62–8.12)	0.219		
Pocket hematoma	1.30 (0.45–3.78)	0.632				0.75 (0.16–3.60)	0.723		

The definitions of parameters are the same as Table 1. CI = confidence interval; HR = harzard ratio

There were 300 patients died during the 4-year follow-up period. After PSM, clinical variables that were significantly associated with all-cause mortality were age, body mass index, coronary artery disease, HF history, CKD, ESRD and anemia in univariate analysis (Table 4). In multivariate Cox regression analysis, age (HR = 1.06, 95% CI, 1.04–1.08, P < 0.001), coronary artery disease (HR = 1.96, 95% CI, 1.39–2.74, P < 0.001), HF history (HR = 1.71, 95% CI, 1.22–2.39, P = 0.002), CKD (HR = 1.96, 95% CI, 1.41–2.71, P < 0.001), ESRD (HR = 2.29, 95% CI, 1.29–4.07, P = 0.005) and anemia (HR = 1.98, 95% CI, 1.43–2.75, P < 0.001) were independent determinants of all-cause mortality (Table 4). Notably, device-related infection was not associated with all-cause mortality (Table 4).

**Table 4:** Univariate and multivariate Cox regression analysis of predictors of all-cause mortality in the overall and matched cohorts

	Overall cohorts					Matched cohorts			
	Univariate analysis		Multivariate analysis			Univariate analysis		Multivariate analysis	
	HR (95% CI)	P value	HR (95% CI)	P value		HR (95% CI)	P value	HR (95% CI)	P value
Age, (years)	1.05 (1.04–1.06)	< 0.001	1.05 (1.03–1.06)	< 0.001		1.05 (1.03–1.07)	< 0.001	1.06 (1.04–1.08)	< 0.001
Male	1.05 (0.84–1.32)	0.678				1.12 (0.83–1.52)	0.451		
Body mass index, (kg/m^2^)	0.94 (0.91–0.97)	< 0.001	0.96 (0.93–0.99)	0.006		0.93 (0.89–0.97)	< 0.001	0.98 (0.94–1.02)	0.235
Hypertension	1.07 (0.83–1.37)	0.612				0.67 (0.49–0.93)	0.016	0.66 (0.47–0.93)	0.017
Diabetes mellitus	1.70 (1.35–2.13))	< 0.001	1.21 (0.95–1.53)	0.128		1.22 (0.90–1.65)	0.206		
Hyperlipidemia	0.86 (0.67–1.09)	0.198				0.76 (0.55–1.05)	0.092	0.77 (0.54–1.08)	0.132
Coronary artery disease	2.44 (1.94–3.08)	< 0.001	1.65 (1.29–2.11)	< 0.001		1.89 (1.38–2.59)	< 0.001	1.96 (1.39–2.74)	< 0.001
Heart failure history	2.14 (1.69–2.70)	< 0.001	1.74 (1.36–2.24)	< 0.001		1.94 (1.42–2.65)	< 0.001	1.71 (1.22–2.39)	0.002
Atrial fibrillation	1.29 (1.03–1.62)	0.028	1.22 (0.97–1.55)	0.096		1.27 (0.94–1.72)	0.119	1.11 (0.81–1.51)	0.514
Cerebrovascular accident	1.48 (1.12–1.96)	0.006	1.23 (0.93–1.64)	0.154		1.17 (0.79–1.74)	0.430		
Chronic kidney disease	3.19 (2.50–4.08)	< 0.001	1.82 (1.39–2.38)	< 0.001		2.05 (1.49–2.81)	< 0.001	1.96 (1.41–2.71)	< 0.001
End-stage renal disease	4.08 (2.92–5.69)	< 0.001	2.37 (1.64–3.43)	< 0.001		2.73 (1.63–4.58)	< 0.001	2.29 (1.29–4.07)	0.005
Anemia	3.52 (2.73–4.54)	< 0.001	2.07 (1.58–2.71)	< 0.001		2.35 (1.70–3.24)	< 0.001	1.98 (1.43–2.75)	< 0.001
Intravenous temporary pacemaker	1.03 (0.78–1.37))	0.827				0.82 (0.56–1.22)	0.326		
Complex procedure	1.04 (0.81–1.33)	0.763				1.11 (0.80–1.52)	0.537		
Pocket hematoma	0.52 (0.21–1.25)	0.142				0.76 (0.28–2.05)	0.587		
Device-related infection	1.26 (0.59–2.66)	0.553				1.32 (0.42–4.16)	0.628		

The definitions of parameters are the same as Table 1. CI = confidence interval; HR = hazard ratio

## Discussion

In this cohort study, the prevalence of CKD in CIED recipients was 45%. The risk of CIED did not differ between CKD patients and non-CKD patient, and CKD was not a risk factor of CIED infection by applying the bundled skin antiseptic preparation strategy before surgery. Patients with CKD had higher cumulative incidences of cardiovascular mortality and all-cause mortality compared to patients without CKD.

### The prevalence of CKD in patients receiving CIEDs

The prevalence of CKD in a decade increased 1.4-fold for stage 3 and 1.7-fold for stage 4 in general population of United States [1]. Cardiovascular diseases, including HF and malignant arrhythmia, remain the leading cause of mortality among CKD patients. A previous study provided evidence for the involvement of the mammalian target of rapamycin pathway that triggers or contributes to ventricular hypertrophy and fibrosis in renal disease [25]. Therefore, patients with CKD are theoretically predisposed to arrhythmic disorders, including asystole, ventricular arrhythmias, and sudden cardiac death [26]. Consequently, the number of CKD patients required CIED implantation also increases gradually. Saad et al. reported that in a cohort with 1235 chronic hemodialysis patients, the prevalence of CIED was 10.5%, including 6.1% with ICD and 4.4% with PPM [5]. Similarly, our previous studies reported that nearly half of CIED recipients had a diagnosis of CKD, and the prevalence of ESRD with chronic hemodialysis in CIED recipients was 6.5%, especially in patients with diabetes [7, 19]. The age of patients in this study (mean age 73 ± 12 years old) was compatible with previous large studies [1, 13, 14]. Therefore, CIED implantation poses a growing and challenging issue to the health of CKD patients, such as CIED infection.

### Risk factors and preventive strategies for CIED Infection in patients with CKD

In the past two decades, the prevalences of CIED implantations, as well as CKD and cardiovascular disease, have increased in the worldwide [27]. However, CIED infection remain a major complication of CIED procedures [9, 12, 17, 28]. The hospitalization for CIED infections continues to increase and is out of proportion to rates of new CIED implants, especially in chronic hemodialysis patients [17, 28]. Previous studies have reported that CKD is an inevitable and non-actionable host-related risk factor for CIED infection and also for poor long-term outcomes [6, 10–18]. According to previous studies, CKD increased 1.5-fold to 4.8-fold risk of CIED infection, and ESRD increased 3.8-fold to 8.7-fold risk of CIED-related infection [10–16]. Moreover, CIED infection increased 2-fold risk of in-hospital mortality, and 5.1-fold risk of 1-year mortality in ESRD patients with chronic hemodialysis compared to patients without hemodialysis [17, 18]. Similar to other risk factors of CIED infection, such as diabetes mellitus, this increased risk of CIED infection in ESRD patients may be attributed to immune dysfunction, presence of indwelling dialysis catheters, and transient bacteremia with repeated hemodialysis treatment [6, 12, 17, 29]. However, in clinical practice, owing to ambiguous local inflammation and infection signs over pacemaker pocket sites, it could be difficult to diagnose device-related infection in ESRD patients in time, resulting in increased morbidity and mortality [16]. Therefore, meticulous clinical follow-up with appropriate pacemaker wound care and patient education deem to be warranted in CKD and ESRD patients receiving CIEDs.

The strongest evidence-based strategy to prevent CIED infection is administration of prophylactic antibiotic before procedures as recommended by current guidelines [30, 31]. However, CIED infection is still not uncommon [17, 28]. We speculated that one of the important reasons is inadequate pre-operative skin antisepsis to sterilize skin flora or minimize the skin flora burden. Da Costa et al. reported that CIED infection-related pathogens are from the skin and pocket of patients, and Lin et al. revealed that the rate of subclinical CIED infection (76.9% with coagulase-negative staphylococci) was 12.0% confirmed by positive bacterial culture of pocket tissues, although subsequent clinical infection did not increase by the presence of subclinical CIED infection [32, 33]. These 2 studies showed that skin flora is responsible for most of the CIED infection. In this study, we demonstrated that after applying the bundled skin antiseptic preparation before surgery, the incidences of clinical major and minor device-related infection in CKD and ESRD patients with dialysis were only 0.1% and 0.8%, respectively, and were not different from those of the patients without CKD (Table 2), consistent with our previous reports [19, 20]. Also, the efficacy of this bundled skin antiseptic preparation strategy did not differ between CKD and non-CKD patients in the subgroup analysis with different comorbidities and risk factors (Fig. 2). Furthermore, traditional procedure-related risk factors of CIED infection, such as hematoma or complex procedure were not found to be risk predictor of CIED infection in this study (Table 3). Accordingly, the bundled skin antiseptic preparation strategy is an effective strategy for decreasing clinical CIED infection in patients with CKD. Patients with CKD and infected CIEDs have been reported to have poor prognosis with incremental long-term mortality, even removal of devices [6, 9, 15, 16]. However, in this study, clinical device-related infection was not a predictor of all-cause mortality in patients with or without CKD (Table 4).

### Long-term clinical outcomes in CKD patients after CIED implantation

**Fig. 4:**
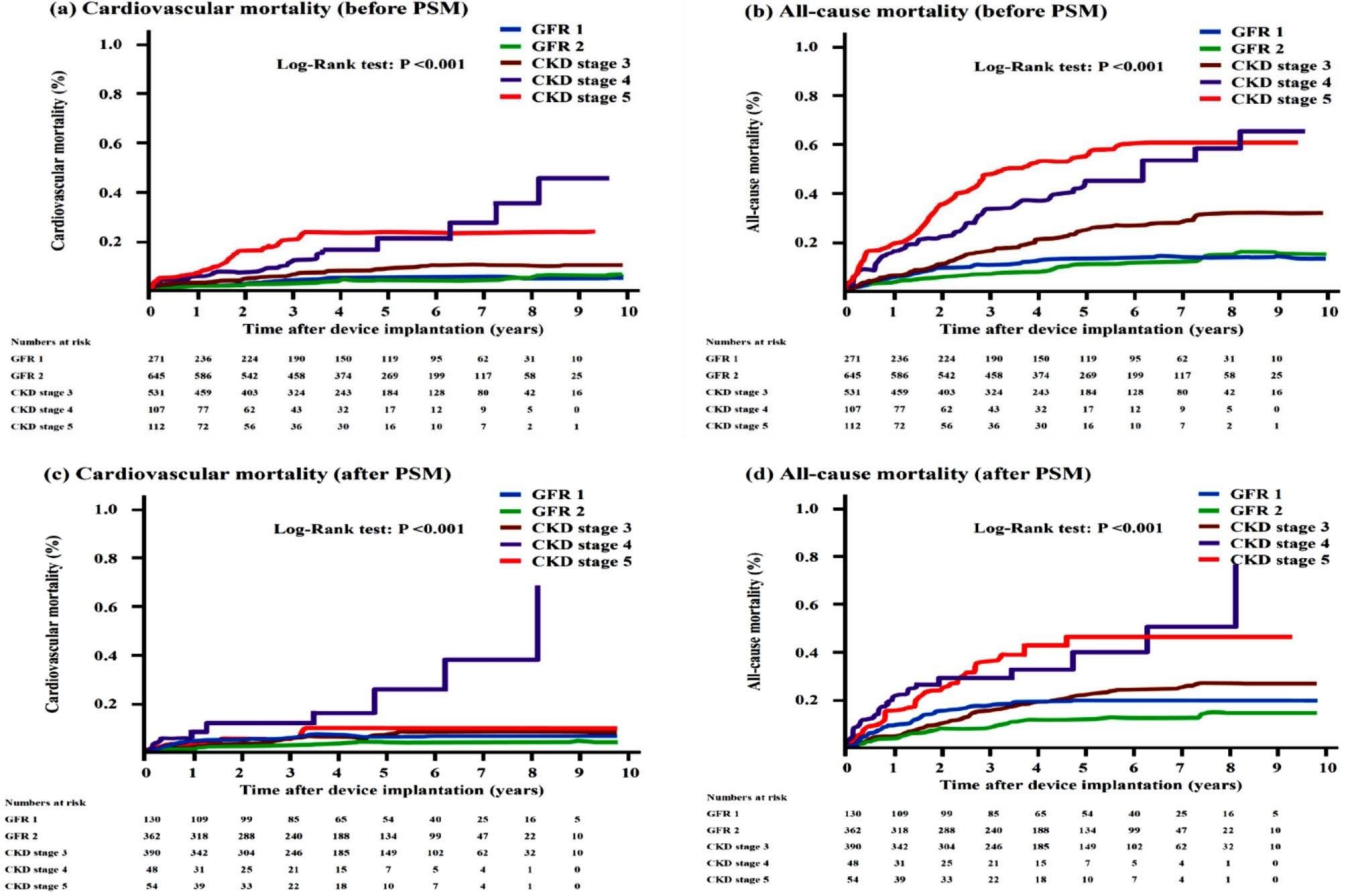
The Kaplan-Meier event-free survival curves of cardiovascular mortality and all-cause mortality between groups with different GFR categories before (Panel **a, b**) and after (Panel **c, d**) propensity score matching. CKD, chronic kidney disease; GFR = glomerular filtration rate; PSM, propensity score matching

Current guidelines identify individuals with CKD as being at increasing risk for cardiovascular disease including coronary artery disease and HF [21]. Vanerio et al. also reported that the risk for all-cause mortality arose from reduced kidney function, after adjustment for other established risk factors [4]. The most common cause of mortality in patients with CKD is sudden cardiac death, leading to around 30% of all-cause mortality in hemodialysis patients [26]. In this study, the incidence of all-cause mortality in patients with CKD is 32% caused by cardiovascular disease, compatible with previous report [26]. Similar to previous studies, this study showed that CKD and ESRD, as well as other traditional risk factors, increase around 2-fold risk of all-cause mortality in patients with CIED implantation (Table 4) [2, 4, 16]. Nevertheless, CKD patients at different stages after CIED implantation, especially in patients with stage 4 and 5, had higher cardiovascular and all-cause mortalities than non-CKD patients (Fig. 4). Therefore, the intensive efforts of guideline-directed medical therapy for patients with CKD are warranted in CKD patients after CIED implantation.

Interestingly, we found that a higher body mass index (BMI) was associated with a significantly lower risk of all-cause mortality in the overall cohort but was not associated with a significantly lower risk of all-cause mortality in the matched cohort (Table 4). This obesity paradox in pacemaker patients was also noted in a large United States National Inpatient database, which showed that obese patients undergoing permanent pacemaker implantation had lower in-hospital mortality compared to non-obese patients [34]. However, the diagnostic discordance between BMI and body fat percentage and misclassification of obesity by BMI in patients with CKD may partly explain the obesity paradox [35].

### Limitation

In this study, some potential limitations existed. First, although this was a retrospective single-center study, the sample size was large. Still, the potential bias inherent to nonrandomized investigations cannot be excluded. However, we performed PSM to minimize the bias between patients with and without CKD. Second, our institute is a tertiary referral center, with a potential for referral bias. Third, the periods of follow-up serum creatinine to assess the stage-to-stage progression of CKD were not regular to be available in all of the patients in this retrospective study.

## Conclusion

The prevalence of CKD in this cohort with CIED recipients was 45%. After PSM, the incidence of clinical CIED infection in patients with CKD was as lower as in patients without CKD after applying the bundled skin antiseptic preparation strategy. During follow-up period, the cumulative incidences of cardiovascular mortality and all-cause mortality were significantly higher in the matched CIED recipients with CKD compared to the matched cohort without CKD. These findings implicate that the intensive efforts of guideline-directed medical therapy for patients with CKD are warranted in CKD patients after CIED implantation.

## Data Availability

The data underlying this article will be shared on reasonable request to the corresponding author.

## References

[1] Coresh J, Selvin E, Stevens LA, Manzi J, Kusek JW, Eggers P (2007). Prevalence of chronic Kidney Disease in the United States. JAMA.

[2] Culleton BF, Larson MG, Wilson PW, Evans JC, Parfrey PS, Levy D (1999). Cardiovascular Disease and mortality in a community-based cohort with mild renal insufficiency. Kidney Int.

[3] GBD Chronic Kidney Disease Collaboration (2020). Global, regional, and national burden of chronic Kidney Disease, 1990–2017: a systematic analysis for the global burden of Disease Study 2017. Lancet.

[4] Tonelli M, Wiebe N, Culleton B, House A, Rabbat C, Fok M (2006). Chronic Kidney Disease and mortality risk: a systematic review. J Am Soc Nephrol.

[5] Saad TF, Ahmed W, Davis K, Jurkovitz C (2015). Cardiovascular implantable electronic devices in hemodialysis patients: prevalence and implications for arteriovenous hemodialysis access interventions. Semin Dial.

[6] Guha A, Maddox WR, Colombo R, Nahman NS, Kintziger KW, Waller JL (2015). Cardiac implantable electronic device Infection in patients with end-stage renal Disease. Heart Rhythm.

[7] Chen HC, Liu WH, Tseng CH, Chen YL, Lee WC, Fang YN, et al. Diabetes increases risk of Cardiovascular events in patients receiving permanent pacemaker: a propensity score-matched cohort study. J Diabetes Res. 2022;6758297. 10.1155/2022/6758297 10.1155/2022/6758297 PMC8979692 35386265

[8] Vanerio G, García C, González C, Ferreiro A. Mortality in patients on renal replacement therapy and permanent cardiac pacemakers. Int J Nephrol. 2014;284172. 10.1155/2014/284172 10.1155/2014/284172 PMC4058238 24977040

[9] Sohail MR, Henrikson CA, Braid-Forbes MJ, Forbes KF, Lerner DJ (2011). Mortality and cost associated with cardiovascular implantable electronic device Infections. Arch Intern Med.

[10] Bloom H, Heeke B, Leon A, Mera F, Delurgio D, Beshai J (2006). Renal insufficiency and the risk of Infection from pacemaker or defibrillator Surgery. Pacing Clin Electrophysiol.

[11] Lekkerkerker JC, van Nieuwkoop C, Trines SA, van der Bom JG, Bernards A, van de Velde ET (2009). Risk factors and time delay associated with cardiac device Infections: Leiden device registry. Heart.

[12] Polyzos KA, Konstantelias AA, Falagas ME (2015). Risk factors for cardiac implantable electronic device Infection: a systematic review and meta-analysis. Europace.

[13] Lin YS, Chen TH, Lin MS, Chen DY, Mao CT, Hsu JT (2016). Impact of chronic Kidney Disease on short-term Cardiac Implantable Electronic device related Infection: a Nationwide Population-based Cohort Study. Med (Baltim).

[14] Birnie DH, Wang J, Alings M, Philippon F, Parkash R, Manlucu J (2019). Risk factors for Infections involving Cardiac Implanted Electronic devices. J Am Coll Cardiol.

[15] Deharo JC, Quatre A, Mancini J, Khairy P, Le Dolley Y, Casalta JP (2012). Long-term outcomes following Infection of cardiac implantable electronic devices: a prospective matched cohort study. Heart.

[16] Hickson LJ, Gooden JY, Le KY, Baddour LM, Friedman PA, Hayes DL (2014). Clinical presentation and outcomes of cardiovascular implantable electronic device Infections in hemodialysis patients. Am J Kidney Dis.

[17] Opelami O, Sakhuja A, Liu X, Tang WH, Schold JD, Navaneethan SD (2014). Outcomes of infected cardiovascular implantable devices in dialysis patients. Am J Nephrol.

[18] Herrmann FEM, Ehrenfeld F, Wellmann P, Hagl C, Sadoni S, Juchem G (2020). Thrombocytopenia and end stage renal Disease are key predictors of survival in patients with cardiac implantable electronic device Infections. J Cardiovasc Electrophysiol.

[19] Chen HC, Chen MC, Chen YL, Tsai TH, Pan KL, Lin YS (2016). Bundled preparation of skin antisepsis decreases the risk of cardiac implantable electronic device-related Infection. Europace.

[20] Chen HC, Lee WC, Chen YL, Tsai TH, Pan KL, Lin YS (2019). Bundled skin antiseptic preparation for complex cardiac implantable electronic device Infection: a propensity-score matching cohort study. J Hosp Infect.

[21] Inker LA, Astor BC, Fox CH, Isakova T, Lash JP, Peralta CA (2014). KDOQI US commentary on the 2012 KDIGO clinical practice guideline for the evaluation and management of CKD. Am J Kidney Dis.

[22] Levey AS, Bosch JP, Lewis JB, Greene T, Rogers N, Roth D (1999). A more accurate method to estimate glomerular filtration rate from serum creatinine: a new prediction equation. Modification of Diet in Renal Disease Study Group. Ann Intern Med.

[23] Nutritional anaemias (1968). Report of a WHO scientific group. World Health Organ Tech Rep Ser.

[24] Austin PC (2009). Balance diagnostics for comparing the distribution of baseline covariates between treatment groups in propensity-score matched samples. Stat Med.

[25] Ritz E (2009). Left ventricular hypertrophy in renal Disease: beyond preload and afterload. Kidney Int.

[26] Turakhia MP, Blankestijn PJ, Carrero JJ, Clase CM, Deo R, Herzog CA et al. Conference Participants. Chronic kidney disease and arrhythmias: conclusions from a Kidney Disease: Improving Global Outcomes (KDIGO) Controversies Conference. *Eur Heart J* 2018;39(24):2314-25. 10.1093/eurheartj/ehy060 10.1093/eurheartj/ehy060 PMC6012907 29522134

[27] Raatikainen MJP, Arnar DO, Merkely B, Nielsen JC, Hindricks G, Heidbuchel H (2017). A decade of information on the Use of Cardiac Implantable Electronic devices and Interventional Electrophysiological Procedures in the European Society of Cardiology Countries: 2017 report from the European Heart Rhythm Association. Europace.

[28] Greenspon AJ, Patel JD, Lau E, Ochoa JA, Frisch DR, Ho RT (2011). 16-year trends in the Infection burden for pacemakers and implantable cardioverter-defibrillators in the United States 1993 to 2008. J Am Coll Cardiol.

[29] Ruiz P, Gomez F, Schreiber AD (1990). Impaired function of macrophage fc gamma receptors in end-stage renal Disease. N Engl J Med.

[30] Baddour LM, Epstein AE, Erickson CC, Knight BP, Levison ME, Lockhart PB, Council on Cardiovascular Surgery and Anesthesia; Council on Cardiovascular Nursing; Council on Clinical Cardiology; Interdisciplinary Council on Quality of Care; American Heart Association. ; American Heart Association Rheumatic Fever, Endocarditis, and Kawasaki Disease Committee; Council on Cardiovascular Disease in Young;. Update on cardiovascular implantable electronic device infections and their management: a scientific statement from the American Heart Association. *Circulation* 2010;121(3):458 – 77. 10.1161/CIRCULATIONAHA.109.192665 10.1161/CIRCULATIONAHA.109.192665 20048212

[31] Sandoe JA, Barlow G, Chambers JB, Gammage M, Guleri A, Howard P (2015). British Society for Antimicrobial Chemotherapy; British Heart Rhythm Society; British Cardiovascular Society; British Heart Valve Society; British Society for Echocardiography. Guidelines for the diagnosis, prevention and management of implantable cardiac electronic device Infection. Report of a joint Working Party project on behalf of the British Society for Antimicrobial Chemotherapy (BSAC, host organization), British Heart Rhythm Society (BHRS), British Cardiovascular Society (BCS), British Heart Valve Society (BHVS) and British Society for Echocardiography (BSE). J Antimicrob Chemother.

[32] Da Costa A, Lelièvre H, Kirkorian G, Célard M, Chevalier P, Vandenesch F (1998). Role of the preaxillary flora in pacemaker Infections: a prospective study. Circulation.

[33] Lin G, Zou T, Dong M, Liu J, Cui W, Tong J (2021). Risk stratifying and prognostic analysis of subclinical cardiac Implantable Electronic devices Infection: insight from traditional bacterial culture. J Am Heart Assoc.

[34] Almani M, Usman M, Qudrat Ullah M, Fatima N, Yousuf M, Edigin E. Impact of obesity on the clinical outcomes of patients undergoing pacemaker insertion during hospitalization: an analysis of the United States National Inpatient Sample. Eur J Prev Cardiol. 2021;28(Issue Supplement1). 10.1093/eurjpc/zwab061.300. zwab061.300.

[35] Lin TY, Lim PS, Hung SC (2017). Impact of misclassification of obesity by body Mass Index on Mortality in patients with CKD. Kidney Int Rep.

